# Separating the Causes of Listening Difficulties in Children

**DOI:** 10.1097/AUD.0000000000001069

**Published:** 2021-06-16

**Authors:** Harvey Dillon, Sharon Cameron

**Affiliations:** 1 Department of Linguistics, Macquarie University, Sydney, Australia; 2 Manchester Centre for Audiology and Deafness, School of Health Sciences, University of Manchester, Manchester, United Kingdom.

**Keywords:** Auditory processing disorders, Listening difficulties, Speech-in-noise deficits, Test battery

## Abstract

Auditory processing disorder, defined here as a deficit in the way sounds are analyzed by the brain, has remained a controversial topic within audiology for decades. Some of the controversy concerns what it is called. More substantively, even its existence has been questioned. That view has likely emerged because there has not been sufficient rigor in determining when difficulty in understanding speech has been the consequence of some type of auditory processing deficit, versus when it is the consequence of a cognitive deficit or a language deficit. This article suggests that the field use the term “listening difficulty” as an umbrella term to indicate a reported deficit in recognizing sounds or understanding speech, one possible cause of which is an auditory processing disorder. Other possible causes are hearing deficits, cognitive deficits, and language deficits. This article uses a plausible, and hopefully noncontroversial, model of speech understanding that comprises auditory processing, speech processing, and language processing, all potentially affected by the degree of attention applied and the listener’s memory ability. In a fresh approach to the construction of test batteries, the stages of the model are linked to tests designed to assess either all or selected parts of the processes involved. For two of the stages, a listener’s performance is quantified as the additional signal to noise ratio that he or she needs to function equivalently to his or her age peers. Subtraction of the deficits revealed by each test enables the contributions of each processing stage to a listening deficit to be quantified. As a further novel contribution, the impact of memory and attention on each test score is quantitatively allowed for, by an amount that depends on each test’s dependence on memory and attention. Attention displayed during the test is estimated from the fluctuations in performance during the test. The article concludes with a summary of the research that must be conducted before the structured tests can be used to quantify the extent to which different potential causes of listening difficulties are responsible for real-life difficulties in an individual child.

*Editor’s Note: The following article is based on the 2018 American Auditory Society Carhart Memorial Lecture and is categorized as a “Point of View” article. As originally described* (Jerger 2000*): “Our second type of new publication, the Point of View article, is a publication with a slant or opinion. This type of article should have a fresh point of view, a clear logic to its propositions, and a clarity of presentation. The article must have a well-reasoned point of view, but the view does not have to be balanced. Our long-term goal for the Point of View article is to stimulate the field’s interest in and to enhance the appreciation of the author’s area of expertise.”*

## INTRODUCTION

This article presents a new conceptual approach to quantifying the extent to which auditory processing disorders (APDs) of various types contribute to children experiencing listening difficulties. Our experience and the data are drawn from children, but the approach may be generalizable to adults. The concepts will be illustrated with examples from recent studies. A major aim of the article is to identify research that may advance the diagnosis and treatment of APDs. The field of APDs is often described as “controversial.” Before dealing with what we think are the substantial issues that must be addressed, we would like to show that the things that are often said to be controversial are actually not significant issues at all.

## FOUR CONTROVERSIES

### Controversy 1: What Do We Call It?

The term central auditory processing disorder (CAPD) has been used since at least 1976 (Sullivan 1976). A consensus conference in 2000 determined that the word Central made the term too limited, because the cochlea also performs auditory processing (Jerger & Musiek 2000). A position statement 5 years later determined that Central was still a useful component because “most definitions of the disorder focus on the central auditory nervous system,” and recommended the term (Central) APD (ASLHA 2005). These three terms are, however, minor variations on a theme compared with the diversity of terms that have been used to describe people who have abnormally poor auditory perception (usually of speech) despite having normal or near–normal-hearing thresholds. These include Central auditory disorder and Disturbances of auditory perception (Myklebust 1954), Central auditory dysfunction (Berry & Blair 1976), Auditory inferiority complex (Byrne & Kerr 1987), Selective dysacusis (Narula & Mason 1988), Auditory disability with normal hearing (Rendell & Stephens 1988), Obscure auditory dysfunction (Saunders & Haggard 1989), Central presbyacusis (Stach et al. 1990), King-Kopetzky syndrome (Hinchcliffe 1992), Auditory dysacusis (Jayaram et al. 1992), and Idiopathic discrimination dysfunction (Rappaport et al. 1993).

More recently than these terms, the terms Auditory neuropathy spectrum disorder (Starr et al. 1996), “Hidden hearing loss” (Schaette & McAlpine 2011), and Cochlear synaptopathy (Kujawa & Liberman 2009) have been used. These three terms describe very specific types of problems in the auditory system, but some researchers have suggested that these problems might be among the causes of the reduced auditory performance that gave rise to all the earlier terms. Hidden hearing loss is also sometimes used as a generic synonym for the 14 terms listed in the previous paragraph.

With the exception of terms that imply a specific site of lesion or mechanism (e.g., cochlear synaptopathy), any term that is widely accepted, and understood in the same way, within and across relevant professions and by parents, should suffice. We will use the term APD, for the reason outlined in the next section.

### Controversy 2: Does APD Exist?

It is clear from the diversity of terms just described that many researchers have, in part independently, come to the view that deficits in auditory perception can occur despite the presence of normal-hearing thresholds. That statement does not imply that the cause of the deficit is necessarily in the auditory system, as will be discussed later in this article. However, the auditory system is enormously complex, even if one just considers the cochlea and the paths and processing from the cochlea to and within the primary auditory cortex. The complexity is even greater if one adds in the connections between the primary auditory cortex and numerous other processing centers elsewhere in the cortex. Is it even conceivable that this complex part of the body is always normally structured at birth, always develops its ability to process complex stimuli in a typical manner during childhood, and never suffers a deterioration in performance later in life? When such a disorder in the auditory processing systems occurs, then what better overall name for it than APD? The question should not be whether APD exists, but rather how often it occurs, how to diagnose it, how to characterize the specific deficit(s) involved, how large the deficits should be before being considered a disorder, and how to remediate it.

### Controversy 3: What Abilities Does APD Encompass?

Any task based on perception of auditory stimuli requires some intelligence, some memory, some attention, some motor functions, and some knowledge of language (even if just to understand the task). If a person is deficient in their ability to perform a task utilizing auditory stimuli, is it useful to label the disorder as an APD if the underlying cause is a deficit in intelligence, memory, attention, motor function, or language? Most position statements by professional societies take the view that it is not helpful to label problems caused by such deficits as APD (ASLHA 2005; BIAP 2007; AAA 2010; CISG 2012). The British Society of Audiology, by contrast, says that APD can have “its origins in …. language, reading, speech, attention, executive function, memory, emotion vision and action” (BSA 2017, p. 6). While a term can be defined any way one wishes, we consider it confusing to include deficits of these types within the definition of APD, though they are extremely important in the wider issue of listening difficulties, which we will discuss in detail. Indeed the Dutch position statement on listening difficulties says that “language or other cognitive processes beyond the traditional auditory system” are the main cause of listening difficulties (De Wit et al. 2017, p. 10).

### Controversy 4: Do Deficits Need to Be Limited to the Auditory Modality?

One view is that a deficit observed when someone responds to auditory stimuli should not be labeled “auditory” unless it can be demonstrated that a similar deficit is not observed in response to stimuli in another sensory modality, such as vision (Cacace & McFarland 1998). The motivation for this view is the desire to not interpret a deficit as an APD if it is caused by a cognitive deficit (Cacace & McFarland 2013). We strongly agree with this goal. However, in this article, we propose a different solution to the problem. The first reason for tackling the problem of auditory specificity in a different way to that proposed by Cacace and McFarland (1998) is that because there are so many differences between auditory and visual stimuli, and between the auditory processing system and the visual processing system, the prospects for designing exactly parallel tasks in these different modalities are poor. The second reason is that it is possible, in principle, for someone to have both an auditory processing deficit of some type and a similar visual processing deficit, with both deficits being unambiguously within their respective neural processing systems. In such a situation, it does not seem appropriate to withhold diagnosis and treatment for the APD, just because the person happens to also have a deficit in the visual processing system.

## A TAXONOMY

**Fig. 1.**
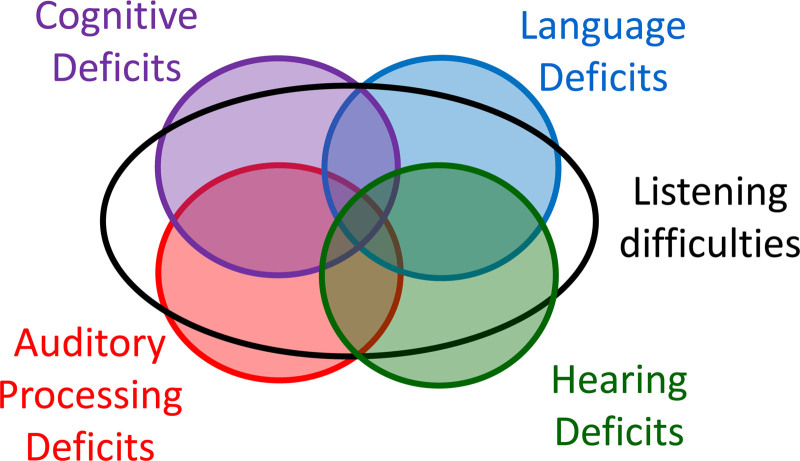
A taxonomy indicating that listening difficulties can be caused by deficits in multiple domains.

The term listening difficulties is increasingly being used to describe children who are observed to have atypical difficulty in understanding speech or other auditory stimuli, often for unknown reasons (Dawes & Bishop 2010; Moore 2012; Ankmnal-Veeranna et al. 2019; Boothalingam et al. 2019; Moore et al. 2020a). It has also been suggested that listening difficulty replace the label auditory processing disorder (Moore 2018). We consider that both terms are useful, but unequivocally should have different meanings. Consistent with Magimairaj and Nagaraj (2018) and others, we view listening difficulties as an umbrella term for observed or self-reported difficulties in understanding or responding to auditory stimuli, especially speech. Auditory processing disorder is also an umbrella term, but it describes a range of specific deficits that cause poorer than normal perception of auditory stimuli. Listening difficulties might be caused by a deficit in auditory processing, but such difficulties might instead be caused by a cognitive deficit, a language deficit, or even a hearing deficit (Dawes et al. 2008; Dawes & Bishop 2009). Figure 1 shows these relationships as a Venn diagram. The overlapping circles indicate that some children may have one, two, three, or even all four of these different broad causes of listening difficulty. Each of the potential deficits can exist independently without necessarily causing listening difficulties. Such a situation could occur if the deficit is mild and is compensated for by the other abilities of the child. The empty spaces in the larger listening difficulties ellipse are not intended to imply that there are causes of listening difficulties that cannot be classified as cognitive, auditory processing, language or hearing in nature, but this might nonetheless be the case. Note that the concept behind this diagram would not change if we were to replace the word “deficit” with “disorder” in each place that it occurs. We regard a disorder as a deficit of sufficiently large magnitude that it represents a problem in real life, but in most instances, the words can be used interchangeably. In this diagram, Hearing deficit is characterized here by elevated hearing thresholds in quiet (i.e., hearing loss), which of course reduces audibility. Whether the hearing deficit is the result of decreased functioning of outer hair cells, inner hair cells, or the synapses to the auditory nerve, more central auditory processes that rely on normal spectral or temporal resolution must also be affected if the peripheral loss has negatively impacted either of these abilities. Speech perception in noise deficits have been linked to hearing threshold elevations that are only minimal (Glyde et al. 2013; Moore et al. 2020b) or are restricted to the high frequencies above 8 kHz (Monson et al. 2019; Motlagh Zadeh et al. 2019; Yeend et al. 2019).

Another term that has been used repeatedly over the past decade is suspected auditory processing disorder. This term is typically used in relation to children with reported listening difficulties who have been brought to a clinic for an APD assessment. However, had the parent taken them to a psychologist for a cognitive assessment or to a speech pathologist for a language assessment, would the same children then be referred to as having suspected cognitive deficits or suspected language deficits, respectively? The term therefore conveys very little information about a group of children who have in common only that someone has suspected they might have some type of APD.

Dawes and Bishop (2009) state that because of the likelihood of there being multiple underlying deficits with overlapping symptoms, clinicians within different professions—each with their own conceptual and diagnostic approach—may diagnose the same child with different labels, such as developmental language disorder, APD, or attention deficit disorder. Our goal, discussed later in this article, is to develop a self-consistent test battery that explicitly allows for different types of deficits affecting speech understanding. In principle, the battery could be administered by an audiologist, a speech pathologist or a psychologist.

Behaviors that could appropriately be described as listening difficulties include difficulty understanding speech in noise or reverberation, difficulties localizing sounds, inconsistent or inappropriate responses to questions, difficulty following rapid speech or instructions, frequent requests for repetition, difficulty maintaining attention, and academic difficulties, including reading, spelling, and learning. It is interesting to note that this list is paraphrased from the AAA (2010) position statement on APD, where they are described as the symptoms of people “at-risk for (C)APD,” and which may reveal “the true functional impact of the (C)APD on the individual’s daily life” (p. 9). There is no inconsistency in us appropriating this useful list to describe listening difficulties rather than APD, as although any of these behaviors may be caused (directly or indirectly) by an APD, they could alternatively be caused by one of the other broad deficit types shown in Figure 1, as the AAA (2010) guidelines acknowledge. This issue leads to the central point of this article: How does the clinician determine which of the domains in Figure 1 are responsible for reported listening difficulties, and how do we uncover the specific deficit(s) within that domain that affect a particular child? Before presenting a model and a methodology for that task, we give an example of why current tests for APD are not sufficient for the task.

## TESTS VERSUS ABILITIES

Tests used in APD assessments are usually intended to assess just one ability. For example, one of the most commonly used APD diagnostic tests is the Dichotic Digits Test (Musiek 1983). By delivering different sounds to each ear at the same time, dichotic tests measure how well a person can attend to the sound in one ear without it being masked by the sound presented to the other ear. The difference in scores between the two ears enables inferences to be made about the functioning of each hemisphere of the cortex, and/or of the corpus callosum connecting the two hemispheres. If the words comprise one digit in each ear, the task is very easy for most people, so the score obtained is usually very close to 100% for both ears, making it hard to detect small asymmetries. To “sensitize” the test, the task is made more difficult by sequentially presenting two digits, or even three digits (Moncrieff & Wilson 2009; Schow et al. 2020) to each ear before asking the listener to freely recall all the digits presented to both ears, or just those for a selected ear. Unfortunately, such a change makes the test more dependent on short-term memory and also on attention. Consequently, and not surprisingly, free recall test scores are moderately correlated with short-term memory (Cameron & Dillon 2020) and highly correlated with measures of attention (Stavrinos et al. 2018). Disconcertingly, free recall scores are highly correlated (*r* = 0.82) with those for a control condition in which the two ears receive exactly the same information, so processes unique to dichotic perception are not involved (Cameron & Dillon 2020). Scores below the normal range in this “dichotic” test can therefore be caused by deficits in dichotic processes, or memory, or attention, considerably weakening their value in differentiating auditory processing deficits from cognitive deficits. This should not be surprising to the field, as the first paper investigating the perception of different digits in each ear was called “The role of auditory localization in attention and memory span” (Broadbent 1954)!

It is extraordinarily hard, if not impossible, to design tests that measure only one ability. A test of speech sentence understanding in competing babble, for example, relies on the listener’s abilities to analyze the incoming mixture of sounds with fine resolution in both time and frequency; separate elements coming from the target from those coming from the competition; identify individual speech sounds from the target talker even when they are partially masked; and use knowledge of semantics, syntax and prosody to fill in the gaps where individual speech sounds could not be perceived at all, or to correct misperceptions of individual sounds. Vermiglio (2014) has proposed that poor speech-in-noise ability is a primary form of deficit that meets the definition of a “clinical entity.” We agree that a speech-in-noise deficit is particularly important for real-life functioning, and is measurable, though the extent of deficit measured in any particular speech-in-noise test will depend on what specific skills the test has been designed to be sensitive to. A measured deficit in speech-in-noise ability certainly tells us that the child will understand less than his or her peers under those same measurement conditions, but tells us nothing about which specific underlying deficits have caused the observed deficit in understanding. The same is true of any other task that involves multiple underlying skills to complete.

The problem persists even when nonspeech sounds are used as the stimulus. The Pitch Pattern Test (Musiek et al. 1980), for example, requires the listener to label (or hum) the pattern of frequencies heard, such as High-Low-High. This apparently simple task requires the listener to be able to discriminate the differences in frequency; store a representation (either as an auditory memory or as a verbal label) while the rest of the sequence is played; retrieve the stored representation in the correct order; assign labels (if not already stored in that form); and give a verbal response. Again, it is not clear what has caused any performance deficit found.

We are pursuing a solution to this problem by using the concept of differential testing to narrow down the range of deficits that could cause scores on a test to be deficient. We will explain this further in the context of an explicit model linking together some abilities involved in understanding speech in acoustically challenging situations.

## MODEL OF SPEECH UNDERSTANDING

**Fig. 2.**
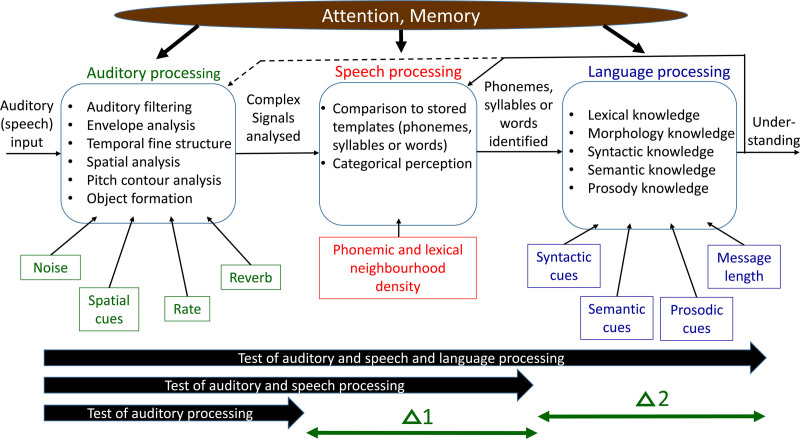
A model of speech understanding involving auditory processing, speech processing, and language processing. The three thick arrows at the bottom indicate tests that assess different components of the model. See text for further details.

Figure 2 shows the model around which we are building a differential test battery. The model is based on the presumption that for speech to be recognized, the incoming sound stream must first be analyzed in various ways. Then individual phonemes, syllables, or words must be identified. Finally, knowledge of language is applied to extract meaning.

The auditory processing that occurs within the first block takes place whether the incoming sounds are speech, nonspeech, or a mixture of the two. The types of auditory processing listed are likely not comprehensive, but are rather concrete examples of how various attributes of sounds can be extracted from the incoming signal. How accurately each of these characteristics of the incoming sound(s) can be measured depends hugely on the specific listening situation. Noise, reverberation, and a fast presentation rate, alone or in combination, may reduce the accuracy with which sounds are analyzed. The presence of spatial cues (e.g., two sounds coming from different directions) likely enhances a listener’s ability to analyze at least one of the simultaneously present sounds. The end result of the first block is the representation of the incoming sound stream as a set of objects, with the acoustic characteristics of those objects analyzed in various ways.

When the sounds of interest are speech sounds, the operations in the second block occur. The representations of the sound (e.g., durations, spectral shape of bursts or longer duration sounds, frequency track of formant transitions) are compared with corresponding stored representations of sounds, and the representation most closely matching the incoming sound is selected. The model is not explicit about whether this identification occurs at the level of the phoneme, the syllable, or the word. How accurately the identification occurs depends on how accurately the first block was able to characterize the incoming signal, and how many candidate sounds in the stored representation have characteristics that are similar to those of the analyzed sound. Thus, accuracy will be higher in quiet than in noise and will be higher when the neighborhood of the analyzed sound has a low phonemic or lexical density (i.e., there are few other similar sounds in the language; Pisoni et al. 1985). The end result of the second block is a set of identified (whether correctly or incorrectly) speech sounds. Having a block dedicated to the identification of speech sounds is consistent with certain cortical lesions that adversely affect the recognition of speech sounds, but not any other sounds (Maffei et al. 2017).

For understanding to be conveyed, the identified sounds must be strung together, and knowledge of the language, or indeed of the world, applied before meaning emerges. When individual speech sounds have been misidentified (or not heard at all), the listener’s knowledge of semantics, syntax, morphology and prosody, plus knowledge of the context in which the communication is taking place, can enable perfect understanding, despite a large proportion of the speech sounds individually not being correctly identified. That is, the listener uses cloze ability (i.e., the ability to apply pre-existing knowledge to other information available to fill in the gaps). How well the listener can do this depends both on the listener’s auditory closure skills and the richness of these language cues in the particular speech stream being attended to.

We do not presume that there is a unidirectional, left-to-right flow of information. Rather feedback occurs to correct earlier misperceptions that become apparent when the message makes no sense, and to predict sounds that are yet to arrive. Such interactions are described in the Ease of Language Understanding model (Rönnberg et al. 2013). The feedback may even extend back to the auditory processing area, as indicated by the dotted arrow in Figure 2.

Finally, many of the processes discussed earlier that are required for speech understanding cannot occur without the application of attention, short-term memory, and working memory. The model is not explicit as to which processes require these cognitive skills to be applied, but we can give two extreme examples. At one extreme, breaking up of sound into frequency bands within the cochlea presumably can occur without the application of any attention or memory. Even here though, we cannot preclude top-down effects involving attention and memory from modifying how it occurs, via modulation of outer hair cell activity by the medial olivo-cochlear bundle (Terreros et al. 2016). At the other extreme, the replacement of an earlier misperceived sound with the correct sound when the error later becomes apparent presumably cannot occur without the application of working memory. We also note that even detection of pure tones (i.e., audiometry), which must be the simplest of auditory tasks, is weakly but significantly correlated with cognitive flexibility (Brännström et al. 2020).

The model presented in Figure 2 is not intended to be either a novel or controversial view of how speech is understood, and the separation of auditory processing from language processing in the context of APD assessment has been proposed before, for example, Richard (2013). The separate components of auditory processing, speech processing, and language processing are consistent with the ventral path of the dual route model of speech perception (Hickok & Poeppel 2007) in which fine acoustic details are analyzed in different areas of cortex than those involved in the recognition of speech sounds (Venezia et al. 2019). The purpose of introducing the model here is to show how its components can be related to tests that can help us uncover the source of listening difficulties in an individual.

## STRUCTURED AUDITORY TESTS

**Fig. 3.**
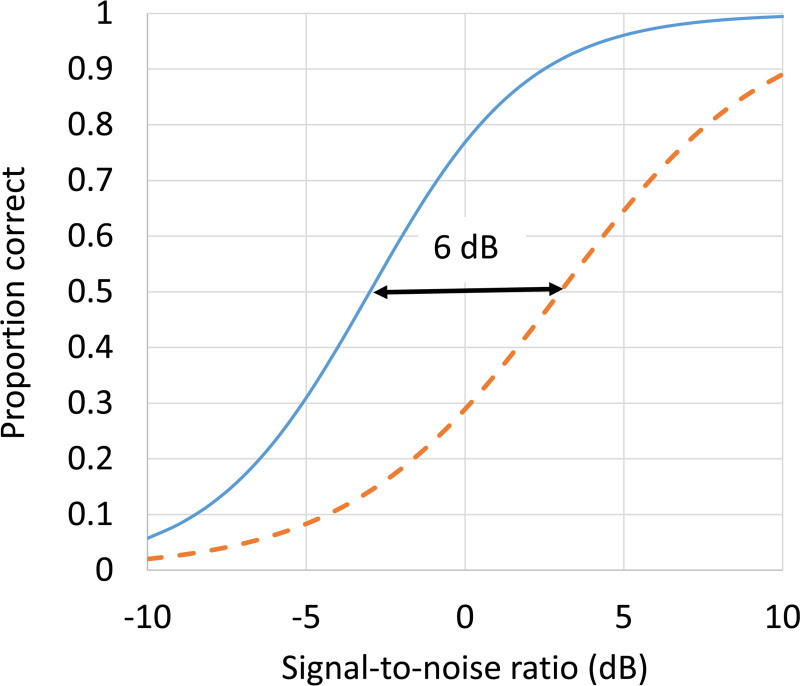
For the top-level test shown in Figure 2 (e.g., perception of meaningful sentences in noise and reverberation), the solid line shows a hypothetical age-appropriate psychometric function, and the dashed line shows the psychometric function for an individual child with a deficit on this task. The arrow shows the magnitude of the deficit, 6 dB, when measured at 50% of items correct.

Suppose there existed a test of speech understanding in noise and reverberation, where the speech material was rich in semantic, syntactic, and prosodic cues. That is, the top-level test in Figure 2. As background noise level increases, the proportion of the message correctly understood by a typical 8-year-old child will drop from 100% at very high signal to noise ratios (SNRs) down to 0% at very poor SNRs, as shown by the solid line in Figure 3. Now suppose the psychometric function for a particular 8-year-old child was like that shown in the dashed line. What can we conclude about the child’s listening ability? The only things we can conclude from this are that the child has a deficit, and that the magnitude of the deficit can be quantified by the extent to which the SNR has to be improved for this child if he/she is to understand as much as his/her age peers. We cannot begin to say whether the cause of this deficit lies in the auditory processing domain, the speech processing domain, the language domain, or the cognitive domain. There may even be contributing deficits in all of these domains.

The moment we add the mid-level speech test, one that requires the identification of speech sounds, but does not require language skills to perform the test, we can start to narrow down where the deficits occur. If the child were to have age-typical scores on this mid-level test, then the most parsimonious explanation of the pair of test results is that when the child was trying to understand speech in the high-level test, he was not able to use age-typical language skills to fill in the gaps in perception caused by the added noise and reverberation. That is, the child has a language deficit, not an auditory processing or speech processing deficit. Conversely, if the deficit in the mid-level test was just as large as in the top-level test, we would infer that the deficit did not lie in the language domain, but rather was in the auditory processing or speech processing domains. In reality, we would interpret the results in a more graded, less dichotomous manner. The difference between the deficit measured with the top-level test and the deficit measured with the second-level test, indicated as Δ2 in Figure 2, will estimate the extent to which language is contributing to the overall speech-in-noise deficit.

Naturally, we can take this approach down to the next level. Suppose we have one or more tests of auditory processing that use nonspeech stimuli, but that require the same types of auditory processing abilities that we routinely apply to analysis of speech sounds. The difference between the deficit in the mid-level test and that in the bottom-level test(s) will indicate the extent to which processes unique to speech sound identification are responsible for the deficit observed in the mid-level test. How this can be done when there are multiple bottom-level tests is discussed later in this section.

The magnitude of the differences between tests at each level will depend on the particular tests chosen, and the performance level chosen at which the two tests are compared. This situation is no different from when a deficit relative to normative data is measured with a single test, as is evident in Figure 3, where the size of the deficit measured depends on the percentage correct chosen to evaluate performance.

The tests at the three levels do not exist yet, but there are some existing tests that come close. At the highest level, context-rich tests of speech understanding can be approximated by context-rich tests of sentence repetition. While in principle a sentence could be repeated by a person without even understanding the language (hence conveying no understanding at all), once a sentence is delivered in significant noise, correct repetition is possible only when understanding is sufficient that language knowledge can be used to correctly fill in the missing words or phonemes masked by the noise. Some tests based on sentences with significant internal context (hence requiring language ability to perform) include the Hearing in Noise test (Nilsson et al. 1994), the Listening in Spatialized Noise Sentences (LiSN-S) test (Cameron & Dillon 2007), and the Bamford-Kowal-Bench Speech-in-Noise test (Etymotic-Research 2005). However, none of these tests are presented in reverberation. Also, none of them use sentences that are long enough to maximize the potential for language knowledge, combined with working memory, to fully apply closure skills at poor SNRs. Further, none of them deliberately aim to maximize internal semantic and syntactic cues. They are also somewhat unrealistic in that recordings used for such tests tend to be articulated with very clear, somewhat slow speech, not typical of everyday communication.

At the middle level, the recently developed Listening in Spatialized Noise Universal (LiSN-U) test (Cameron et al. 2020) uses nonsense syllables common to most of the world’s languages, in a consonant-vowel-consonant-vowel format, as target stimuli. The test thus measures the ability to identify speech sounds, in both a spatialized and nonspatialized context, but does not depend on the language ability of the listener, at least vocabulary, semantic, and syntactic abilities. The test does not even require that the listener speaks any particular language, though they must be able to speak some language. We are currently developing a more advanced version of the test, which also measures the impact of reverberation on speech understanding.

The bottom-level test, or set of tests, is the most difficult to conceptualize. It is also the most important because it is only at this level that we can identify the specific auditory processing deficits responsible for reduced understanding of speech in challenging listening situations. There are, of course, already many clinical tests that use nonspeech stimuli. These include tests of gap detection (Keith 2000; Musiek et al. 2005), order perception based on frequency or duration differences (Musiek 1994), binaural masking level difference (Goldstein & Stephens 1975), and the ability to count a rapid series of impulses (Schow, Reference Note 1). There is an even greater range of tests that have been used in psychoacoustic research such as discrimination of small changes in frequency, intensity, or duration; sensitivity to temporal fine structure presented monaurally or binaurally; and detection of modulation in intensity, spectral shape, or both at once. Each of these (by design) examines only a very limited range of auditory processing abilities and typically uses very simple stimuli to do it. The processing involved in analyzing the characteristics of a sound as complex as speech may be more complex than that tested by most of these existing tests. The acoustic signal conveying target speech sounds and competition has to be analyzed in multiple ways simultaneously, and must be done rapidly, without the high degree of conscious attention that is typically possible in a clinical or psychoacoustic test of auditory processing ability.

The low-level test will therefore require either a new design, that uses complex stimuli and requires several types of complex auditory processes to perform, or it will need to be a combination of tests, each testing one aspect of auditory processing, but using more realistically complex stimuli, with the results combined in an evidence-based manner. As an example, a gap detection test is commonly incorporated in APD test batteries. However, all available clinical gap detection tests measure “within-channel gap detection,” meaning that the stimuli preceding and following the gap have the same frequency content (Keith 2000; Musiek et al. 2005; Schow, Reference Note 1). It is easy to point to the relevance of gap detection for speech perception, but gaps critical to speech perception are surrounded by two sounds that differ in their frequency spectra. This task, which has been referred to as between-channel gap detection, has larger gap detection thresholds than for within-channel gap detection (Phillips & Smith 2004). Between-channel gap detection requires the auditory system to compare when activity ceases in nerve fibers responsive to one frequency range, relative to when activity later commences in a different set of nerve fibers. This must require more complex processing than is required for within-channel gap detection where “all” that has to be detected is a momentary reduction of activity in any of the nerve fibers activated by the stimulus. Certainly, performance on the two tasks is not highly correlated in the normal-hearing adult population (Phillips & Smith 2004).

Assuming that it will require more than one low-level test to adequately measure the range of auditory processes that affect how complex signals like speech are recognized, how should the results of the separate tests be combined to assess the overall impact of these abilities on speech perception? The current approach when a battery of APD tests is used is to consider only the worst one or two results in determining whether the child has APD. The AAA (2010) recommendation is to diagnose APD if two test scores are more than 2 SDs below the mean for children of that age, or one test score is more than 3 SDs below the mean. It seems more useful to consider a more graded criterion. For example, a child that was 1.9 SDs below the mean on six different auditory processing abilities would likely have greater difficulty in real life than one who was 2.1 SDs below on two abilities, but completely normal on the other four. Yet, the first child would meet the diagnosis for APD, but not the second! How then should the scores from multiple low-level tests be combined? The scores can be weighted and added, not necessarily in a linear manner. A nonlinear formula might be needed to ensure that above-average scores for some abilities do not inappropriately cancel out the perhaps greater impact of below-average scores for other abilities. The weights, and the type of nonlinearity required can, in principle, be deduced from large data sets relating the scores on the tests to some outcome measure that requires all the auditory processing abilities assessed in the low-level tests. While the outcome measure could be a parental rating of listening ability, or a sentence-based test of speech understanding, both of these measures can be affected by things other than auditory processing ability. Consequently, a more direct outcome measure against which the importance of each low-level ability can be assessed would be the mid-level speech sound perception task in noise and reverberation described earlier. We currently have under development tests of between-channel gap detection and spectrotemporal resolution (broadly similar to that used by Bernstein et al. 2013), but there is no certainty that these tests will be either necessary or sufficient to adequately capture the range of auditory processes needed to analyze speech before recognition of its constituent phonemes or syllables.

Whatever its constituent parts, how would a weighted average score be used in practice? First, when expressed as a *z* score relative to the distribution of such scores in the population, it would provide an overall estimate of the basic auditory processing ability of a child insofar as it affects perception of speech. A sufficiently low score would indicate the appropriateness of performing remediation for whatever individual deficits were the largest. Second, subtraction of this score from the score for the mid-level test (also expressed in *z* score units) would provide an estimate of the additional deficits (or superior abilities) that the child showed in recognizing speech sounds, given the child’s abilities in auditory processing. A deficit in that difference score, indicating poor speech sound recognition relative to the listener’s auditory processing ability for nonspeech sounds, would presumably indicate that remediation should be focused on auditory training involving speech sounds rather than on some more basic auditory processing skill.

## ALLOWING FOR THE EFFECTS OF COGNITIVE ABILITIES ON TEST SCORES

The preceding section outlined how deficits in the language and speech processing domains might be separated from deficits in the auditory processing domain. What about deficits in cognition, particularly in attention and memory?

We propose that the impact of memory and attention on auditory test scores (whether using speech or nonspeech stimuli) can be allowed for in a statistical manner, similar to how age is usually allowed for. Suppose that scores obtained on some APD test are analyzed using multilinear regression with age and measured memory and attention as predictor variables. We can then estimate the expected APD test scores as:

$${\text{APD}}_{\text{estimated}}=a+b.\text{age}+c.\text{memory}+d.\text{attention}\text{,}$$ (1)

where *a*, *b*, *c*, and *d* are the coefficients that minimize prediction error when the multilinear regression is carried out. For any individual, the difference between the observed APD score and the score expected on the basis of age, memory, and attention reflects the contribution that the underlying APD ability (plus random measurement error) makes to the observed score. Equation 1 can be applied to quantify this difference as follows:

$$\begin{array}{l} {\text{APD}}_{\text{observed}}-{\text{APD}}_{\text{estimated}}=\\ \;\;\;\;\;\;\;\;\;\;\;\;\;\;\;\;\;\;\;\;\;\;\;\;\;\;\;{\text{APD}}_{\text{observed}}-a-b.\text{age}-c.\text{memory}-d.\text{attention} \end{array}$$ (2)

Equation 2 indicates that our best estimate of the underlying APD ability is obtained by subtracting from the test score the expected effects of age, memory, and attention on the task. Correcting scores for age is routinely done in APD assessments. Although corrections for age may not usually be expressed in the quantitative manner made explicit by Equations 1 and 2, the effect is the same. Age is included as a term here to show that correcting for memory and attention is no different in principle to correcting for age. Such corrections seem appropriate if performing the APD task places significant demands on memory or attention, such that variations in memory or attention ability directly affect APD test scores.

Before an APD test score can be corrected for memory or attention, we must first decide what type of memory and attention should be measured. The appropriate types of cognitive ability are, of course, the ones that most strongly affect performance on the test. Take, again, for example, the commonly used dichotic digits test. In a sample of 93 children brought to clinics for APD assessment, the free recall total score on the Dichotic Digits difference test was significantly more highly correlated with a forward digit span memory test (*r* = 0.60) than with a reverse digit span test of working memory (*r* = 0.38) (Cameron & Dillon 2020). That is, simple short-term memory was more predictive of performance on the dichotic free recall task than reverse digit span. This seems plausible, as the dichotic free recall task directly involves hearing four digits, storing them, and repeating them, with no need to perform mental operations on the digits while they are stored. It is likely, however, that some APD tests might place more demands on working memory (involving significant executive functioning) than on simple short-term memory. In principle, there is no problem in extending Equation 1 by simultaneously allowing for more than one type of memory or attention ability or additional cognitive abilities like fluid reasoning.

Allowing for attention is potentially more complex. Should we allow for variations in sustained attention, switching attention, divided attention, or executive attention? If one type of attention is selected, should it be measured with auditory stimuli (as the most relevant sensory modality) or with visual stimuli (to minimize the likelihood that a true APD impacts on the measurement of attention)? Given that the attention a child brings to a task likely depends on how motivating they find the task, how can we be sure that the level of attention measured during the attention test was also applied during the APD test if the tasks differed in how well they engaged the child?

**Fig. 4.**
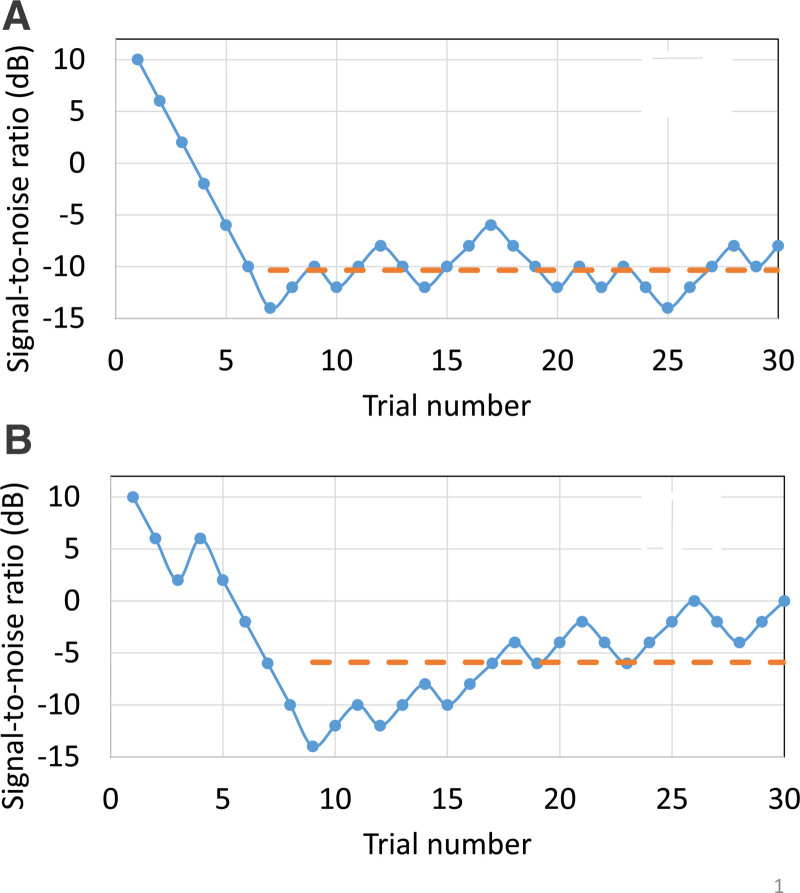
Adaptive tracks (hypothetical) for a child (A) who maintains a high level of attention throughout the task, and (B) whose attention appears to fluctuate and decrease as the measurement progresses. In each case, the dashed line shows the average SNR during the measurement phase of the track. SNR indicates signal to noise ratio.

A possible answer to these dilemmas is to directly estimate the attention, or at least fluctuations in attention, that the child displays during the APD test. In an adaptive test comprised of equally difficult test items, it is reasonable to expect a well-behaved adaptive track like that shown in Figure 4A. After a preliminary “hunting” phase, the SNR (or other stimulus parameter being adjusted to control difficulty) varies by only a limited amount around the average value during the measurement phase (commencing at trial 7 in Fig. 4). The SNRs during the measurement phase are averaged to produce the test result, such as a speech reception threshold (SRT). If the step size is appropriate relative to the width of the psychometric function underlying performance on the test, then wrong answers to two or three trials in a row will cause the next trial to be so easy it should be perceived correctly, and two or three correct answers in a row will make the next trial so difficult it is unlikely to be perceived correctly.

If tracks like that in Figure 4A are commonly obtained with a test, then what can we make of a track like that in Figure 4B? Initially, it looks like the child is headed toward a similar SRT to that of the first track, averaging a SNR of about −10 dB. However, performance then deteriorates as the track swings upward. A possible explanation is that the child’s attention on the task decreased. For this child, the average SNR during the measurement phase is around −6 dB, as indicated by the dashed line. We can quantify the variations during the measurement phase by measuring the standard deviation in the SNRs presented. In track A, the standard deviation is only 2.0 dB, whereas, in track B, it is 4.1 dB. We hypothesize that this metric estimates the inconsistency of attention with which the child has performed the task. Intuitively, larger standard deviations should be associated with poorer performance, as reported by Moore (2012). This is because fluctuating attention should always cause someone to perform worse than they would, were they to maintain consistently high attention throughout the test. As suggested by Equation 2, the measured SRT could be approximately corrected for attention by subtracting the standard deviation of the fluctuations times some unknown factor (coefficient *d* in Equation 2). Although the correction based on track fluctuations is motivated by the wish to compensate for variable attention, it may have another beneficial effect. Sometimes, a gradual improvement in performance is visible during the measurement phase of the adaptive track. Such tracks likely indicate that the child was actually getting better at the task during the measurement. If so, a simple average across the track underestimates the child’s true ability. Because the gradual improvement contributes to track variability, subtraction of some proportion of the variability from the mean score will result in a test result closer to the child’s final performance than to their initial performance.

**Fig. 5.**
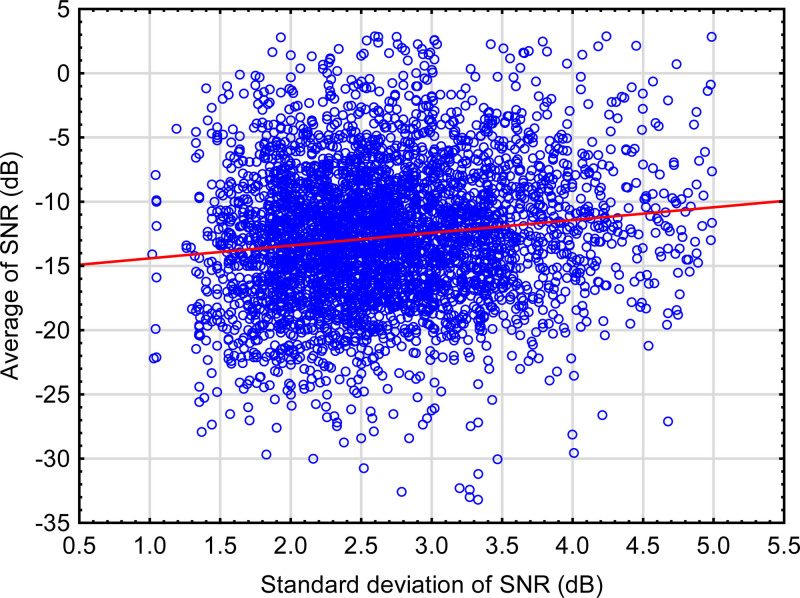
Average of the SNRs during the measurement phase (i.e., the SRT) versus the standard deviation of the SNRs during the measurement phase for the speech-in-noise subtest of the Sound Scouts online hearing test. The solid line shows the linear regression of the SRT on the standard deviation. SNR indicates signal to noise ratio; SRT, speech reception threshold.

The size of *d* in Eqs. (1) and (2) can be estimated by comparing the mean SNR during the measurement phase (i.e., the SRT) to the standard deviations of the adaptive track for typical data. Figure 5 shows this relationship for the speech-in-noise subtest of the Sound Scouts online hearing test (Dillon et al. 2018). The solid line shows the regression of the mean on the standard deviation. The slope of this line is 0.994. That is, on average, for every 1 dB increase in the standard deviation of the adaptive track during the measurement phase, the measured SRT also increases by 1 dB. While the 95% confidence interval around this slope is very narrow, from 0.990 to 0.998, because of the large number of data points, there is no certainty that the same slope would apply to other data sets. Nonetheless, it does seem plausible that when adaptive tracks are very variable, the best performance that the child is capable of will be better than the mean value by an amount similar to the standard deviation of the track itself. These data are based on responses from 4793 children aged from 4.1 to 17.9 years of age (mean 8.7 years). Note that there is no information known about the hearing or auditory processing ability of these children, other than what is measured by the Sound Scouts app itself. For this reason, and because of the wide age range, there is a wide range of SRT values for any particular value of the standard deviation. The relationship between SRT and the variability of the track has emerged despite this large interparticipant variability.

While the idea of correcting tracks for attention has not previously been proposed, variability in adaptive tracks has previously been linked to attention and hearing ability. Moore et al. (2010) found that as track variability increased, performance worsened on the psychoacoustic ability being measured. Their interpretation was that track variability was an indicator of the child’s attention generally, and that poor attention was responsible for the child’s listening problems in real life: “Our research suggests that APD in children is primarily a result of poor engagement with sounds rather than impaired hearing” (Moore et al. 2010, p. e389). Our interpretation is more limited: variable attention during the task has adversely affected the measurement, so the score obtained is not a true indicator of the child’s auditory ability, were they to be giving the task sustained high attention. Variable attention evident during the adaptive measurement may or may not be indicative of variable attention to auditory stimuli in real life, and the relationship likely depends on how engaging the auditory test is.

Note that the above discussion concerns correcting scores from auditory tests for the effects of variable attention during the test. To completely understand where a child’s real-life listening difficulties stem from, it may also be necessary to have a separate measure of attention, thought to be representative of the attention that the child applies in real life, but that discussion is outside the scope of this article.

## RESEARCH NEEDS

This article has proposed a test structure that we believe will enable the cause(s) of listening difficulties to be identified in individual children. The remaining part of this article identifies what research is needed before such a structure could be implemented clinically. The mid-level test is not discussed as we have already developed a prototype, which we have called the Language-Independent Speech in Noise and Reverberation test. It is an enhanced version of the LiSN-U test that uses nonsense syllables common to most languages (Cameron et al. 2020). The new test also assesses the impact of reverberation on speech sound understanding and allows for the impact of fluctuations in attention on performance.

### Top-Level Test

A top-level test, rich in language cues and presented with adaptive SNR in a simulated acoustically challenging environment, must be developed. Unfortunately, a language-specific version must be developed in whatever language the children to be tested normally communicate. (Fortunately, all the other tests in the battery can then be independent of language.) Optionally, derivatives of the top-level test could also be developed with any one of the language cues removed. For example, the words could be rearranged into syntactically correct, but semantically unrelated sentences, or into semantically related word lists with no syntax. Those children for whom the deficit in speech understanding was primarily caused by language could be presented with these speech-in-noise subtests to determine which aspects of language the children were inadequately using to fill in the gaps. Manipulation of the level of semantic redundancy in a speech-in-noise task is not a new suggestion (Elliott 1979; Lagacé et al. 2010, 2011) but does not appear to have been acted upon in tests available clinically.

### Lower Level Tests

Significant effort will be required to develop lower-level tests that challenge the auditory system in ways that tests using simple stimuli may not. The auditory processing required for the task should have complexity commensurate with the types of auditory processing needed to understand speech, but without using any speech sounds. Yet, the task itself should be simple and presented in an engaging way, so that children as young as possible can be tested reliably.

### Normative and Reliability Data

Every test must have normative and test-retest reliability data. Normative data should be expressed as a continuous function of age rather than for discrete age groups of arbitrary span. Doing so increases the accuracy of the age-dependent means and avoids discontinuities or even nonmonotonicity, as age increases from one age category to the next (Tomlin et al. 2014). Reliability data must be obtained on the types of populations to which the test is to be applied.

### Relationships Between Tests

The relationships between results on the low-, mid-, and top-level tests must be measured in both typically developing children and in children with reported listening difficulties. It would be instructive to also include children with low proficiency in the language used in the test (e.g., listening in their second language). The relationship of each level of test to questionnaire-based ratings of real-life listening difficulty, and to measured cognitive abilities must also be known, with due allowance made for the reliability of each of these measures, as well as for any potential biases in scores based on questionnaires. These relationships will provide the information needed to apportion the causes of listening difficulties in individual children and to correct test scores for the effects of cognitive abilities. The strength of the relationships must be interpreted in relation to the measured reliability of each test in similar populations. An observed weak correlation coefficient of, say 0.4, can actually indicate a strong underlying correlation of, say 0.8, once the impact of measurement error has been taken into account by using known reliability coefficients for the two measures. This technique has systematically been applied in development of the Multiple Auditory Processing Assessment battery (Schow, Reference Note 1). Such corrections can totally change the interpretation of how closely two abilities are related.

### Construct Validity

For any APD test designed to measure some specific ability, test results should be compared to those for another test, constructed as differently as possible, that is also designed to measure the same specific ability (Dawes & Bishop 2009). Only when the two tests are highly correlated (after allowing for random measurement error) can one be convinced that the test is measuring the specific ability in question.

### Test Score Cut-Offs

Understanding the true relationships between test scores and either speech perception in realistically challenging environments or rated real-life listening ability will also enable us to know, for the first time, how important each low-level auditory processing ability is to the complex task of understanding speech in challenging listening situations. We can also use these relationships to systematically determine which tasks are important enough to include in a test battery, and how far below normal an individual auditory processing test score should be before it causes a significant problem for real-life communication. In principle, the answer likely depends on the child’s other auditory processing, cognitive, and language abilities. That is, the cut-off score at which we consider there is a problem need not arbitrarily be set at 2 SDs below the mean on every test.

### Efficient Test Structure

There is no point presenting several tests of specific abilities to a listener if other test results already make it unlikely that any of the specific tests not yet administered will reveal a deficit. A test battery structure and set of rules are needed to guide which tests, in which order, are administered to any individual child. The presenting characteristics, preferably quantified as questionnaire scores, could also be incorporated into these rules. Research studies are then needed to assess how often, and under what circumstances, the rules produce suboptimal outcomes.

### Deficit-Specific Interventions

Interventions must be developed and evaluated, and existing interventions must be evaluated, to determine their real-life consequences. It is inadequate to show that a deficit-specific intervention causes scores on a test originally used to diagnose the deficit to increase after training. This can too easily be caused by statistical regression to the mean. Even if the increase is real, the improvement may be in a skill so specific to the test task that it does not generalize to real-life listening. As far as we are aware, the deficit-specific intervention we have developed for spatial processing disorder is the only training for APD that has been shown in a blinded randomized trial, albeit small, to have benefits for listening in real-life situations (Cameron et al. 2012).

### Determining Causation

Determining relationships between tests involves analyzing correlations, or their big brother and sister, multilinear regression and structural equation modeling. Unfortunately, these techniques can never unambiguously determine the direction of causation. Intervention studies are needed where just one ability is trained (e.g., a specific auditory processing ability, in a way that minimizes the likelihood of also training memory or attention). If all other correlated abilities are measured before and after training, then any changes or lack of change can be used to infer the presence and direction of causation.

### Electrophysiological Tests

Eventually, we need to develop standardized, clinically viable, electrophysiological tests of each auditory processing ability. These tests are unlikely to involve responses evoked by simple unchanging stimuli, but rather tests that reflect the ability to discriminate different stimuli. Such tests may enable testing to be carried out on children younger than is likely to be possible with behavioral testing.

## ACKNOWLEDGMENTS

We would like to thank Jorg Buchholz for his insightful comments on an earlier draft.
